# Trends and Disparities in Coronary Artery Disease and Obesity‐Related Mortality in the United States From 1999–2022

**DOI:** 10.1002/edm2.70010

**Published:** 2024-10-27

**Authors:** Mushood Ahmed, Hira Javaid, Aimen Shafiq, Zain Ali Nadeem, Areeba Ahsan, Abdullah Nofal, Raheel Ahmed, Mahboob Alam, Marat Fudim, Gregg C. Fonarow, Mamas A. Mamas

**Affiliations:** ^1^ Department of Medicine Rawalpindi Medical University Rawalpindi Pakistan; ^2^ Department of Medicine Allama Iqbal Medical College Lahore Pakistan; ^3^ Department of Medicine Dow University of Health Sciences Karachi Pakistan; ^4^ Department of Medicine Foundation University Medical College Islamabad Pakistan; ^5^ Department of Medicine Services Institute of Medical Sciences Lahore Pakistan; ^6^ National Heart & Lung Institute Imperial College London London UK; ^7^ Department of Cardiology Royal Brompton Hospital London UK; ^8^ Division of Cardiology The Texas Heart Institute, Baylor College of Medicine Houston Texas USA; ^9^ Department of Medicine Duke University Medical Center Durham North Carolina USA; ^10^ Duke Clinical Research Institute Durham North Carolina USA; ^11^ Ahmanson‐UCLA Cardiomyopathy Center, Division of Cardiology University of California Los Angeles Los Angeles California USA; ^12^ Keele Cardiovascular Research Group, Centre for Prognosis Research Keele University Stoke‐On‐Trent UK

**Keywords:** CDC WONDER, coronary artery disease, mortality, obesity

## Abstract

**Background:**

Almost half of the US adult population has obesity, which predisposes to atherosclerosis and can lead to poor prognosis in coronary artery disease (CAD). We aim to identify CAD and obesity‐related mortality trends among adults in the United States stratified by age, sex, race and geographical location.

**Methods:**

The CDC‐WONDER database was used to extract death certificate data for adults aged ≥ 25 years. Crude mortality rates (CMR) and age‐adjusted mortality rates (AAMRs) per 100,000 persons were calculated, and temporal trends were described by calculating annual percent change (APC) and the average APC (AAPC) in the rates using Joinpoint regression analysis.

**Results:**

From 1999 to 2022, a total of 273,761 CAD and obesity‐related deaths were recorded in the United States. The AAMR increased consistently from 1999 to 2018 (APC: 4.3, 95% confidence interval (CI): 3.4–4.9) and surged thereafter till 2022 (APC: 11.4; 95% CI: 7.7–19.1). During the COVID‐19 pandemic (2020–2022), AAMR almost doubled that of the rest of the study period. Additionally, the AAMR for males was nearly twice that of females. Non‐Hispanic (NH) Blacks or African Americans displayed the highest AAMR, followed by NH Whites, Hispanic or Latino, and other NH populations. AAMRs showed minimal variation by census regions. Rural areas exhibited a higher AAMR (AAMR: 5.9, 95% CI: 5.8–5.9) than urban areas (AAMR: 4.4, 95% CI: 4.4–4.5).

**Conclusions:**

We observed increasing trends in CAD and obesity‐related deaths throughout the study period reaching a peak during the COVID‐19 pandemic.

## Graphical abstract

Obesity is a key risk factor for coronary artery disease (CAD) and can lead to increased risk of mortality. This study explored the mortality trends in individuals with concomitant CAD and obesity over two decades. The mortality rate of CAD and obesity gradually increased from 1999 to 2018, and a peak was observed during the COVID‐19 pandemic when the mortality rates almost doubled. Males had an almost double age‐adjusted mortality rate (AAMR) that of females. Rural areas had higher AAMR than urban areas.

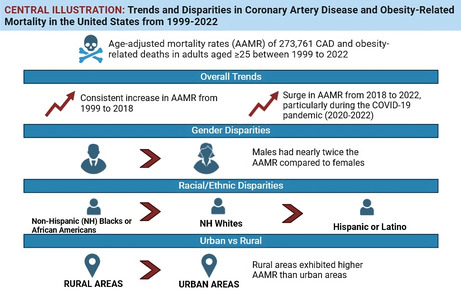

## 1 Introduction

Coronary artery disease (CAD) and its associated complications remain among the primary causes of morbidity and mortality [1]. For individuals over 35, CAD ranks as the third most common cause of mortality in the United States [2]. Obesity is a key risk factor for CAD. It increases the risk of atherosclerosis and worsens the prognosis of CAD [3]. Several cardiovascular risk factors, such as insulin resistance, dyslipidaemia and hypertension, are exacerbated by obesity and enhance the likelihood of developing CAD [4].

According to estimates, approximately 49% of people are overweight or obese [3]. Although the relationship between obesity and CAD is well‐established, there is a notable variation in mortality rates from CAD among obese people in the United States across various demographic and geographical locations [5, 6]. Comprehending these disparities is crucial in discerning high‐risk cohorts and formulating focused therapies to alleviate the influence of obesity on mortality associated with CAD. In this study, the CDC WONDER database was analysed from 1999 to 2022 to evaluate CAD‐related mortality trends in adults with obesity in the United States stratified by age, sex, race and geographical location differences to identify populations at heightened risk.

## 2 Methods

### 2.1 Study Setting

We utilized data provided by the National Center for Health Statistics (NCHS) available through the Centers for Disease Control and Prevention Wide‐Ranging Online Data for Epidemiologic Research (CDC‐WONDER) Database to analyze annual mortality trends for individuals over 25 years of age from 1999 to 2022. The data are annually updated using death certificates of US residents, which contain both the underlying cause of death and demographic information. Using the final Multiple Cause of Death Public Use Record and International Classification of Diseases, 10th Revision (ICD‐10) codes: I20–I25 for CAD and E66.0‐E66.9 for obesity, we identified death certificates listing these conditions as underlying or contributing causes of death. These ICD‐10 codes have been used in prior studies [7, 8]. Notably, our study did not necessitate approval from an Institutional Review Board (IRB) as it relied on anonymised and publicly available data. Furthermore, the research strictly adhered to the STROBE guidelines [9].

### 2.2 Data Extraction

The comprehensive dataset used for analysis included a wide array of demographic variables, that is, sex, race/ethnicity, age groups, region, state and urban–rural classification. Sex categories included males and females. Race/ethnicity groups were delineated as non‐Hispanic (NH) white, NH Black or African American, NH others (NH Asian or Pacific Islander, NH American Indian or Alaska Native, etc.) and Hispanics or Latinos. For age stratification, age was divided into the following categories: 25–34, 35–44, 45–54, 55–64, 65–74, 75–84 and 85 years and older. Furthermore, trends in mortality from CAD and obesity were evaluated based on state‐specific variations, different census regions in the United States (Northeast, Midwest, South and West), and specific county‐level urbanisation classifications (rural: micropolitan and noncore regions; urban: large central metro, large fringe metro, medium metro and small metro regions) [10].

### 2.3 Statistical Analysis

CAD and obesity‐related crude mortality rates (CMRs) and age‐adjusted mortality rates (AAMRs) were obtained. AAMR controls for the population's variation in age distribution, allowing data comparison, and was computed using the direct method of adjustment using the 2000 standard population [11]. The Joinpoint Regression Program (Joinpoint version 5.1.0, National Cancer Institute) was employed to analyse age‐adjusted mortality trends from 1999 to 2022 [12]. This program leverages serial permutation tests to examine repeated time trends and identify up to a single inflection point where the rate of change of mortality is statistically significantly different. Subsequently, the program calculates the weighted average annual percent change (APC) for each time segment in the AAMR, along with corresponding 95% confidence intervals (CIs). An APC estimate was calculated to indicate an increase or decrease if the slope of the trend significantly differed from zero; otherwise, the trend was denoted as stable. A pairwise comparison was performed to determine whether the differences in APCs were significantly different across various subgroups (sex, race, census regions and urbanisation). A statistically significant trend change was indicated by a *p* value of < 0.05.

## 3 Results

### 3.1 Annual Trends for CAD and Obesity‐Related AAMR

**FIGURE 1:**
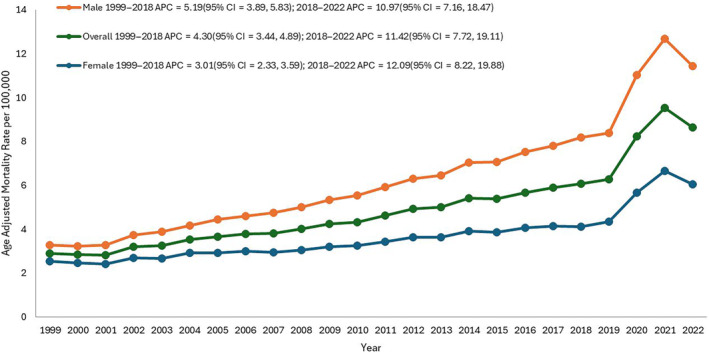
Overall and sex‐stratified coronary artery disease and obesity‐related age‐adjusted mortality rates (AAMRs) per 100,000 individuals in the United States, 1999–2022.

**FIGURE 2:**
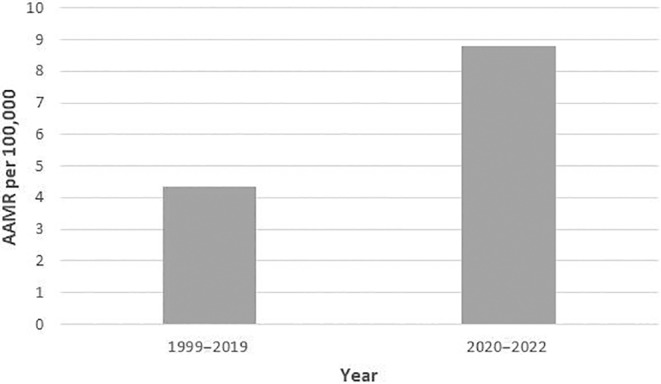
Comparison of age‐adjusted mortality rates (AAMRs) per 100,000 individuals before and during COVID‐19 pandemic.

A total of 273,761 deaths (Table S1) were identified among adults (≥ 25 years of age) between 1999 and 2022 attributed to CAD and obesity. The AAMR for CAD and obesity‐related deaths in adults was 2.9 in 1999 and increased to 8.6 in 2022. The AAMR exhibited a steady rise from 1999 to 2018 (APC: 4.3; 95% CI: 3.4–4.9; *p* = 0.001), followed by a substantial increase from 2018 to 2022 (APC: 11.4; 95% CI: 7.7–19.1; *p* < 0.000001, Table S2, Figure 1). The AAMR peaked during the COVID‐19 pandemic, with mortality rates from 2020 to 2022 more than doubling compared to the rest of the study period (Table S3, Figure 2).

### 3.2 CAD and Obesity‐Related AAMR Stratified by Sex

The AAMR for men was consistently higher than that of women over the study period. In 1999, the AAMR for adult men stood at 3.3 (95% CI: 3.2–3.4), increasing to 8.2 in 2018 (APC: 5.2; 95% CI: 3.9–5.8; *p* = 0.01, Table S3), followed by a notable rise to 11.4 in 2022 (APC: 10.9; 95% CI: 7.2–18.5; *p* < 0.00001). Similarly, the AAMR for adult women in 1999 was 2.5 (95% CI: 2.5–2.7), progressively rising to 4.1 in 2018 (APC: 3.0; 95% CI: 2.3–3.6; *p* < 0.00001), followed by a sudden increase to 6.1 in 2022 (APC: 12.1; 95% CI: 8.2–19.9; *p* < 0.00001). The pairwise comparison indicated a significant difference between the mortality of male and female individuals.

### 3.3 CAD and Obesity‐Related AAMR Stratified by Race/Ethnicity

Regarding race/ethnicity, the highest AAMRs were observed among NH Black or African American, followed by NH White, Hispanic or Latino, and other NH populations (NH American Indian or Alaska Native and NH Asian or Pacific Islander).

The NH Black or African American group exhibited an AAMR of 4.7 in 1999, which increased to 7.6 in 2018 (APC: 3.6; 95% CI: 2.6–4.5; *p* = 0.0001), followed by a further increase to 11.9 in 2022 (APC: 13.8; 95% CI: 8.7–23.9; *p* < 0.000001, Table S4).

The mortality rate for NH White also demonstrated a steadily increasing trend from 2.9 to 6.5 from 1999 to 2018 (APC: 4.5; 95% CI: 3.8–5.1; *p* = 0.001), with a rise to 9.2 in mortality rate from 2018 to 2022 (APC: 11.2; 95% CI: 7.7–18.3; *p* < 0.000001).

**FIGURE 3:**
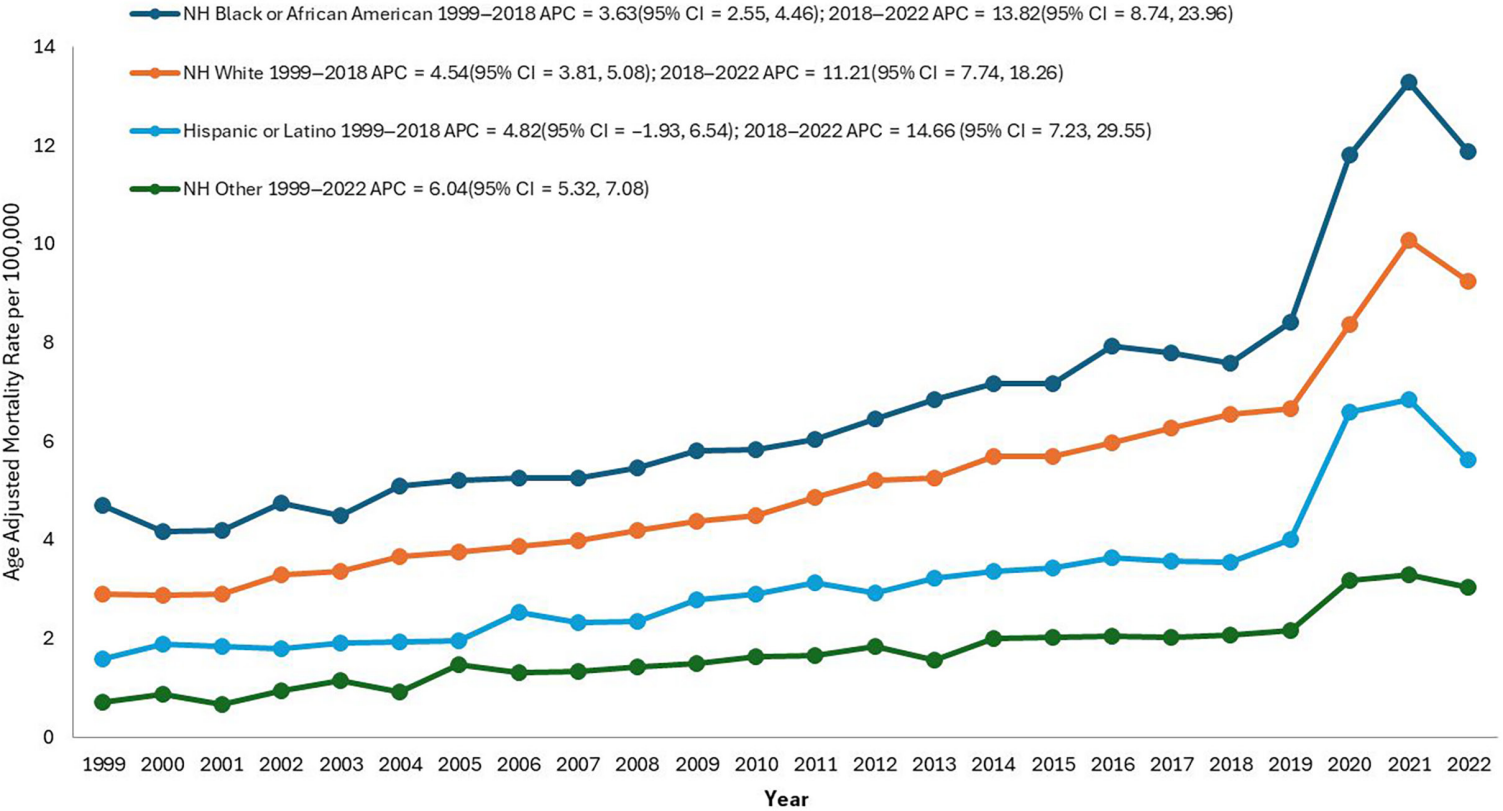
Coronary artery disease and obesity‐related age‐adjusted mortality rates (AAMRs) per 100,000 individuals stratified by race in the United States, 1999–2022.

For Hispanics or Latinos, there was an increase in the AAMR from 3.5 to 6.5 from 1999 to 2018 (APC: 4.8; 95% CI: −1.9 to 6.5; *p* < 0.06). From 2018, a sharp rise in the mortality rate was observed, which continued until 2022 (APC: 14.7; 95% CI: 7.2–29.6; *p* < 0.000001, Figure 3). The AAMR for Hispanic or Latino at the end of the study period was 5.6.

Among NH's other populations, the AAMR displayed a fluctuating increase from 0.7 to 3.0 from 1999 to 2022 (APC: 6.0; 95% CI: 5.3–7.1, *p* < 0.000001).

### 3.4 CAD and Obesity‐Related AAMR Stratified by Geographical Region

#### 3.4.1 State

Between 1999 and 2020, Vermont had the highest AAMR of 16.0. Oklahoma exhibited the second‐highest AAMR of 8.9, while Alabama reflected the lowest AAMR at 2.3 (Table S5). Subsequently, a discernible increase in AAMR was documented during the 2021–2022 period for several states. States that ranked in the upper 90th percentile for CAD and obesity‐related mortalities in 2021–2022 included Vermont, South Carolina, Oklahoma, Wisconsin and Wyoming. Conversely, states within the lower 10th percentile in the same period included New Jersey, Hawaii, Massachusetts, Alabama and Virginia.

#### 3.4.2 Census Region

The AAMR exhibited minimal variation across census regions. In 1999, the AAMR for the Midwest region was 2.9, experiencing a steady increase to 8.9 by 2022 (APC: 5.5; 95% CI: 5.0–6.2; *p* < 0.000001). The Northeast region had an AAMR of 2.9 in 1999, which gradually climbed to 5.6 in 2017 (APC: 4.4; 95% CI: 2.8–4.9; *p* = 0.017) and increased to 7.7 in 2022 (APC: 8.2; 95% CI: 5.7–14.3; *p* < 0.000001). The AAMR for the South region showed a stable rise from 2.7 to 5.7 between 1999 and 2018 (APC: 4.0; 95% CI: 3.2–4.8; *p* < 0.000001, Table S6 and Figure S1), followed by a notable surge, resulting in an AAMR of 9.2 by 2022 (APC: 15.3; 95% CI: 10.9–23.4; *p* < 0.000001). Similarly, the West region's AAMR was 3.3 in 1999, increased steadily to 5.9 in 2018 (APC: 3.5; 95% CI: 2.5–4.1; *p* = 0.003), and rose significantly to 8.1 by 2022 (APC: 10.8; 95% CI: 6.7–19.3; *p* < 0.000001). Overall, during the period of study spanning from 1999 to 2020, it was observed that the Midwest region exhibited the highest AAMR at 5.6. Meanwhile, from 2021 to 2022, the South region recorded the highest AAMR, at 9.7. Throughout both study periods, the Northeast region consistently demonstrated the lowest AAMR, registering values of 4.81 and 7.9 for 1999–2020 and 2021–2022 respectively.

#### 3.4.3 Urban–Rural

**FIGURE 4:**
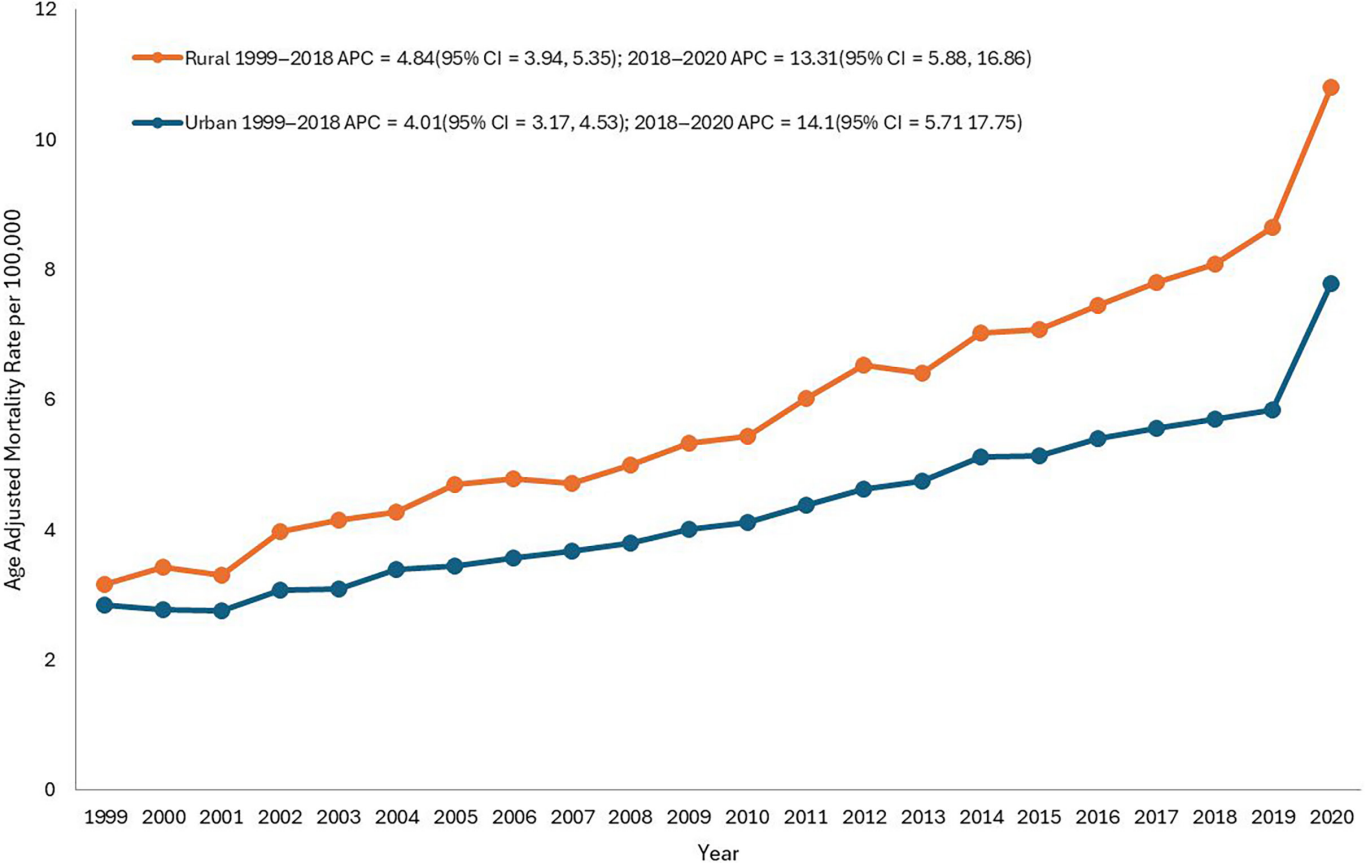
Coronary artery disease and obesity‐related age‐adjusted mortality rates (AAMRs) per 100,000 individuals stratified by urbanisation in the United States, 1999–2020. *Data for urbanisation AAMRs was unavailable for 2021–2022 based on CDC final multiple cause of death data.

From 1999 to 2020, rural areas displayed consistently higher CAD and obesity‐related AAMRs than urban areas, with overall AAMRs of 5.9 and 4.4 respectively. Specifically, the AAMR in rural areas steadily increased from 2.8 in 1999 to 5.7 in 2018 (APC: 4.84; 95% CI: 3.9–5.4; *p* = 0.0004, Table S7 and Figure 4), followed by a steep incline to 7.8 in 2020 (APC = 13.3; 95% CI: 5.9–16.9; *p* < 0.000001). Similarly, the AAMR for urban areas consistently increased from 3.2 in 1999 to 8.1 in 2018 (APC: 4.0; 95% CI: 3.2–4.5; *p* = 0.002), and a significant rise to 10.8 was observed in 2020 (APC = 14.1; 95% CI: 5.7–17.8, *p* < 0.000001). Data for AAMR was unavailable for 2021–2022 based on CDC data. The difference in mortality of urban–rural areas had a significant difference as indicated by the pairwise comparison (Table S8).

### 3.5 CAD and Obesity‐Related CMR Stratified by 10‐Year Age Groups

In the analysis stratified by age groups, the highest CMRs were observed in the 65–74 and 75–84 age categories, followed by the 45–54 and 85+ age groups. Conversely, the 25–34 and 35–44 age groups displayed the lowest CMRs (Table S9 and Figure S2).

## 4 Discussion

Our analysis of CAD and obesity‐related mortality trends among adults in the United States from 1999 to 2022 reveals several significant disparities among different patient groups. A rise in obesity is associated with a higher burden of linked disorders, involving cardiovascular mortality. Thus, a rising public health priority is discovering and treating the underlying causes of obesity and associated inequities among various populations.

From 1999 to 2018, there was a gradual increase in the AAMR for CAD and obesity. However, there was a substantial spike from 2018 to 2022. This increase can be linked to several factors. In the United States, the incidence of obesity has been rising, which has increased the number of people at risk for CAD [13]. Mortality rates may be impacted by differences in healthcare quality and availability in various places and periods, especially if healthcare advancements are not uniformly dispersed. Importantly, our findings validate greater relative increases in obesity‐associated CVD mortality compared to overall CVD mortality in the COVID era. The significant spike in mortality rates from 2018 to 2022 can be largely attributed to the impact of the COVID‐19 pandemic. COVID‐19 posed a higher risk to individuals with pre‐existing conditions, including CAD and obesity, causing severe respiratory and cardiovascular complications and leading to higher mortality rates. Additionally, healthcare systems worldwide were overwhelmed, resulting in delays in routine medical care, screenings and treatments for chronic conditions. Behavioural changes induced by the pandemic, such as increased stress, reduced physical activity and unhealthy eating habits due to lockdowns and social restrictions, further exacerbated obesity and related health issues.

The AAMR for males was nearly twice as high as for females, which might be explained by several factors: men and women differ in the distribution of body fat and how their bodies react to obesity, which can affect the progression of CAD [14]. Furthermore, males suffer from CAD‐related deaths at a higher rate than women, even in the absence of obesity. Men are often less inclined than women to practice preventative health behaviours [15], and they may use healthcare services at lower rates. Oestrogen is believed to have a protective effect against heart disease [14], which may partially explain the lower mortality rates in women until they reach menopause [13, 15].

We report significant differences in AAMRs between various racial and ethnic groups. NH Black or African Americans showed the highest AAMR. This finding probably reflects the disproportionate impact of obesity on Black people, who have been found to have the greatest estimated prevalence of obesity of any racial group in the United States [6]. This finding may be explained by the increased incidence of diabetes and obesity and restricted access to high‐quality healthcare services in these population groups [16]. Comorbid diseases including diabetes and hypertension, healthcare availability and socioeconomic variables also play an important role.

Regional differences in AAMRs were substantial. The Midwest had the highest AAMR, which may have been driven by increased obesity rates, food preferences and socioeconomic variables that affect health outcomes. Additionally, prior research has repeatedly demonstrated that, in general, health outcomes are worse in rural areas, mostly as a result of less access to healthcare. Hence reduced healthcare access, a lower socioeconomic standing, and limited opportunities for physical exercise along with nutritious dietary choices are all potential causes of higher death rates observed in non‐metropolitan locations [17].

Among obese adults, those between the ages of 65–74 and 75–84 had the highest CMR for CAD and obesity. Age‐related risks may assist in clarifying this tendency [18]. The combined impact of risk factors such as long‐term obesity, diabetes, and hypertension places elderly people at increased risk for CAD [19]. The treatment of CAD is made more challenging in this age group by the higher likelihood of several concomitant diseases. Additionally, delays in diagnosis and treatment are among the healthcare utilisation patterns observed in the elderly, and they can affect mortality rates.

The rising trends observed in our pooled analysis of CAD‐related mortality in obese adults underscore the critical need for focused public health initiatives. Important suggestions consist of promoting a balanced diet, consistent exercise [20], and weight control that can help lower the prevalence of obesity and the CAD risk that follows. Improving access to high‐quality healthcare is essential for the early identification and treatment of CAD, especially in the observed high‐risk groups and underserved rural locations. Interventions have to be customised to meet the unique requirements of high‐risk populations, such as men, NH Black or African Americans, people living in the Midwest, people living in rural regions and senior citizens. Understanding these unique disparities and the need to have a tailored approach to them can help guide public health interventions and policies to mitigate the impact of obesity on CAD mortality.

Some limitations must be acknowledged when interpreting our findings. The study relied on data from death certificates, and co‐existing pathologies may have influenced our results. We do not have data regarding patients' baseline characteristics, baseline cardiovascular risk and previous history of cardiovascular events which could have influenced our findings. As the CDC WONDER does not contain information about the potential confounders such as socioeconomic status, healthcare access or comorbid conditions, we were not able to analyse their impact on mortality rates. Additionally, changes in diagnostic methods, coding practices and increased survival rates for patients with co‐existing obesity and CAD could contribute to higher prevalence rates, thereby resulting in an elevated observed mortality rate.

## 5 Conclusion

An increasing trend of CAD and obesity‐related deaths in adults was observed throughout the study period. The highest mortality was exhibited by males and NH Black or African Americans, residents of the Midwest, rural areas and individuals aged 65–74 and 75–84 years. To lower the mortality rates from CAD and obesity in the United States, better healthcare attitudes and lifestyle changes should be encouraged in these high‐risk populations as well as in rural areas. This will help inform public health interventions and policies that aim to lessen the impact of obesity on CAD mortality.

## Author Contributions

Conceptualisation, data curation and project administration were carried out by M.A. Supervision was carried out by M.F., M.A.M. and G.C.F. Formal analysis of data was carried out by M.A., H.J. and Z.A.N. Formal analysis, methodology and software were carried out by A.N., Z.A.N., H.J., A.A., and M.A. Writing the original draft was carried out by M.A., A.S., H.J., A.N. and Z.A.N. Writing, reviewing and editing were carried out by M.A.M., M.F., R.A. and G.C.F. Visualisation and validation were carried out by M.F., M.A.M., R.A. and G.C.F.

## Ethics Statement

The authors have nothing to report.

## Consent

The authors have nothing to report.

## Conflicts of Interest

Dr. Fonarow reported receiving personal fees from Abbott, Amgen, AstraZeneca, Bayer, Boehringer Ingelheim, Cytokinetics, Eli Lilly, Johnson & Johnson, Medtronic, Merck, Novartis and Pfizer outside the submitted work. Dr. Fudim reported receiving personal fees from Alleviant, Ajax, Alio Health, Alleviant, Artha, Audicor, Axon Therapies, Bayer, Bodyguide, Bodyport, Boston Scientific, Broadview, Cadence, Cardioflow, Cardionomics, Coridea, CVRx, Daxor, Deerfield Catalyst, Edwards LifeSciences, Echosens, EKO, Feldschuh Foundation, Fire1, FutureCardia, Galvani, Gradient, Hatteras, HemodynamiQ, Impulse Dynamics, Intershunt, Medtronic, Merck, NIMedical, NovoNordisk, NucleusRx, NXT Biomedical, Orchestra, Pharmacosmos, PreHealth, Presidio, Procyreon, ReCor, Rockley, SCPharma, Shifamed, Splendo, Summacor, SyMap, Verily, Vironix, Viscardia and Zoll; and receiving grants from the National Institutes of Health, Doris Duke, outside the submitted work. No other disclosures were reported.

## Supporting information


Appendix S1.

## Data Availability

All data generated or analysed during this study are included in this article. Further inquiries can be directed to the corresponding author.

## References

[1] F. Sanchis‐Gomar , C. Perez‐Quilis , R. Leischik , and A. Lucia , “Epidemiology of Coronary Heart Disease and Acute Coronary Syndrome,” Annals of Translational Medicine 4, no. 13 (2016): 256. 27500157 10.21037/atm.2016.06.33 PMC4958723

[2] J. E. Dalen , J. S. Alpert , R. J. Goldberg , and R. S. Weinstein , “The Epidemic of the 20th Century: Coronary Heart Disease,” American Journal of Medicine 127, no. 9 (2014): 807–812. 24811552 10.1016/j.amjmed.2014.04.015

[3] T. M. Powell‐Wiley , P. Poirier , L. E. Burke , et al., “Obesity and Cardiovascular Disease: A Scientific Statement From the American Heart Association,” Circulation 143, no. 21 (2021): 973, 10.1161/CIR.0000000000000973. PMC8493650 33882682

[4] C. J. Lavie , R. V. Milani , and H. O. Ventura , “Obesity and Cardiovascular Disease,” Journal of the American College of Cardiology 53, no. 21 (2009): 1925–1932. 19460605 10.1016/j.jacc.2008.12.068

[5] R. Alizadehsani , A. Khosravi , M. Roshanzamir , et al., “Coronary Artery Disease Detection Using Artificial Intelligence Techniques: A Survey of Trends, Geographical Differences and Diagnostic Features 1991–2020,” Computers in Biology and Medicine 128 (2021): 104095. 33217660 10.1016/j.compbiomed.2020.104095

[6] Z. Raisi‐Estabragh , O. Kobo , J. H. Mieres , et al., “Racial Disparities in Obesity‐Related Cardiovascular Mortality in the United States: Temporal Trends From 1999 to 2020,” Journal of the American Heart Association 12, no. 18 (2023): e028409. 37671611 10.1161/JAHA.122.028409 PMC10547286

[7] K. A. Wilmot , M. O'Flaherty , S. Capewell , E. S. Ford , and V. Vaccarino , “Coronary Heart Disease Mortality Declines in the United States From 1979 Through 2011: Evidence for Stagnation in Young Adults, Especially Women,” Circulation 132, no. 11 (2015): 997–1002. 26302759 10.1161/CIRCULATIONAHA.115.015293 PMC4828724

[8] A. Qamar , D. Abramov , V. Bang , N. W. Chew , O. Kobo , and M. A. Mamas , “Has the First Year of the COVID Pandemic Impacted the Trends in Obesity‐Related CVD Mortality Between 1999 and 2019 in the United States?,” International Journal of Cardiology Cardiovascular Risk and Prevention 21 (2024): 200248. 38590764 10.1016/j.ijcrp.2024.200248 PMC10999992

[9] E. von Elm , D. G. Altman , M. Egger , et al., “The Strengthening the Reporting of Observational Studies in Epidemiology (STROBE) Statement: Guidelines for Reporting Observational Studies,” Journal of Clinical Epidemiology 61, no. 4 (2008): 344–349. 18313558 10.1016/j.jclinepi.2007.11.008

[10] D. D. Ingram and S. J. Franco , “2013 NCHS Urban‐Rural Classification Scheme for Counties,” Vital and Health Statistics 2 166 (2014): 1–73. 24776070

[11] R. N. Anderson and H. M. Rosenberg , “Age Standardization of Death Rates: Implementation of the Year 2000 Standard,” National Vital Statistics Reports 47, no. 3 (1998): 16–20. 9796247

[12] Joinpoint Regression Program, Version 5.1.0 , Statistical Methodology and Applications Branch, Surveillance Research Program (Bethesda, MD: National Cancer Institute, 2024).

[13] Y. Wang , M. A. Beydoun , J. Min , H. Xue , L. A. Kaminsky , and L. J. Cheskin , “Has the Prevalence of Overweight, Obesity and Central Obesity Levelled Off in the United States? Trends, Patterns, Disparities, and Future Projections for the Obesity Epidemic,” International Journal of Epidemiology 49, no. 3 (2020): 810–823. 32016289 10.1093/ije/dyz273 PMC7394965

[14] M. R. Meyer and M. Barton , “Estrogens and Coronary Artery Disease,” in Advances in Pharmacology (Amsterdam, The Netherlands: Elsevier, 2016), 307–360. 10.1016/bs.apha.2016.05.003 27451102

[15] K. Yahagi , H. R. Davis , E. Arbustini , and R. Virmani , “Sex Differences in Coronary Artery Disease: Pathological Observations,” Atherosclerosis 239, no. 1 (2015): 260–267. 25634157 10.1016/j.atherosclerosis.2015.01.017

[16] A. Natarajan , M. Gould , A. Daniel , R. Mangal , and L. Ganti , “Access to Healthcare in Rural Communities: A Bibliometric Analysis,” Health Psychology Research 11 (2023): 90615. 38089642 10.52965/001c.90615 PMC10712557

[17] G. K. Singh , R. E. Azuine , M. Siahpush , and M. D. Kogan , “All‐Cause and Cause‐Specific Mortality Among US Youth: Socioeconomic and Rural‐Urban Disparities and International Patterns,” Journal of Urban Health: Bulletin of the New York Academy of Medicine 90, no. 3 (2013): 388–405. 22772771 10.1007/s11524-012-9744-0 PMC3665977

[18] S. Saadatagah , M. G. Varughese , and V. Nambi , “Coronary Artery Disease Risk Prediction in Young Adults: How Can We Overcome the Dominant Effect of Age?,” Current Atherosclerosis Reports 25, no. 6 (2023): 257–265. 37195598 10.1007/s11883-023-01106-1

[19] M. V. Madhavan , B. J. Gersh , K. P. Alexander , C. B. Granger , and G. W. Stone , “Coronary Artery Disease in Patients ≥ 80 Years of Age,” Journal of the American College of Cardiology 71, no. 18 (2018): 2015–2040. 29724356 10.1016/j.jacc.2017.12.068

[20] V. M. Conraads , N. Pattyn , C. De Maeyer , et al., “Aerobic Interval Training and Continuous Training Equally Improve Aerobic Exercise Capacity in Patients With Coronary Artery Disease: The SAINTEX‐CAD Study,” International Journal of Cardiology 179 (2015): 203–210. 25464446 10.1016/j.ijcard.2014.10.155

