# Supplementary material for: Trends and Disparities in Coronary Artery Disease and Obesity‐Related Mortality in the United States From 1999–2022
Source: Endocrinol Diabetes Metab. 2024 Oct 27;7(6):e70010. doi: 10.1002/edm2.70010 (PMC11512744; doi:10.1002/edm2.70010)
Supplement: Supplementary file 1 — Appendix S1. [file EDM2-7-e70010-s001.docx]

**Supplementary appendix**

**Supplemental Table 1: Coronary artery disease (CAD) and obesity related deaths, Stratified by Sex and Race in the United States, 1999 to 2022**

|  | Deaths | | | | | |
| --- | --- | --- | --- | --- | --- | --- |
| Year | **Female** | **Male** | **NH White** | **NH Black or African American** | **NH other** | **Hispanic or Latino** |
| 1999 | 2464 | 2704 | 4113 | 804 | 46 | 187 |
| 2000 | 2425 | 2731 | 4113 | 733 | 59 | 226 |
| 2001 | 2404 | 2830 | 4163 | 763 | 52 | 240 |
| 2002 | 2757 | 3298 | 4821 | 894 | 74 | 244 |
| 2003 | 2752 | 3517 | 5015 | 862 | 100 | 272 |
| 2004 | 3081 | 3831 | 5520 | 996 | 84 | 293 |
| 2005 | 3157 | 4171 | 5782 | 1060 | 139 | 323 |
| 2006 | 3279 | 4425 | 6023 | 1098 | 132 | 424 |
| 2007 | 3290 | 4663 | 6257 | 1125 | 137 | 416 |
| 2008 | 3466 | 5029 | 6675 | 1197 | 155 | 448 |
| 2009 | 3724 | 5453 | 7102 | 1311 | 167 | 554 |
| 2010 | 3777 | 5759 | 7375 | 1352 | 188 | 588 |
| 2011 | 4106 | 6273 | 8019 | 1447 | 203 | 657 |
| 2012 | 4404 | 6848 | 8742 | 1559 | 233 | 669 |
| 2013 | 4540 | 7121 | 8972 | 1681 | 203 | 758 |
| 2014 | 4929 | 7903 | 9826 | 1815 | 273 | 844 |
| 2015 | 5005 | 8090 | 9948 | 1872 | 281 | 912 |
| 2016 | 5310 | 8669 | 10508 | 2098 | 306 | 1001 |
| 2017 | 5502 | 9236 | 11210 | 2112 | 309 | 1018 |
| 2018 | 5555 | 9807 | 11787 | 2109 | 330 | 1063 |
| 2019 | 5932 | 10114 | 12046 | 2357 | 352 | 1225 |
| 2020 | 7919 | 13540 | 15443 | 3388 | 528 | 2046 |
| 2021 | 9253 | 15775 | 18345 | 3736 | 633 | 2247 |
| 2022 | 8568 | 14375 | 17046 | 3359 | 591 | 1860 |

NH, non-Hispanic.

**Supplemental Table 2: Annual percent change (APC) of coronary artery disease and obesity-related age-adjusted mortality rates per 100,000 in the United States, 1999 to 2022**

| **Year Interval** | **APC (95% CI)** |
| --- | --- |
| **Overall** |  |
| 1999-2018 | 4.3036 (3.4467 - 4.8996) |
| 2018-2022 | 11.4280 (7.7237 - 19.1151) |
| **Male** |  |
| 1999-2018 | 5.1903 (3.8984 - 5.831) |
| 2018-2022 | 10.9716 (7.1695 - 18.4754) |
| **Female** |  |
| 1999-2018 | 3.0137 (2.3364 - 3.5947) |
| 2018-2022 | 12.0941 (8.225 - 19.8863) |
| **NH White** |  |
| 1999-2018 | 4.5457 (3.8157 - 5.0848) |
| 2018-2022 | 11.2195 (7.7422 - 18.2602) |
| **NH Black or African American** |  |
| 1999-2018 | 3.6343 (2.5771 - 4.464) |
| 2018-2022 | 13.8237 (8.7414 - 23.9612) |
| **NH other** |  |
| 1999-2022 | 6.0402 (5.3273 - 7.088) |
| **Hispanic or Latino** |  |
| 1999-2018 | 4.8238 (-1.9341 - 6.5479) |
| 2018-2022 | 14.6697 (7.238 - 29.5589) |
| **Rural areas** |  |
| 1999-2018 | 4.8454 (3.9427 - 5.3501) |
| 2018-2020 | 13.3121 (5.8829 - 16.8633) |
| **Urban areas** |  |
| 1999-2018 | 4.0153 (3.1779 - 4.5369) |
| 2018-2020 | 14.1668 (5.7179 - 17.7543) |
| **Age*** |  |
| **25-34 years** |  |
| 1999-2022 | 5.0794 (4.305 - 6.0602) |
| **35-44 years** |  |
| 1999-2022 | 5.0720 (4.6542 - 5.5716) |
| **45-54 years** |  |
| 1999-2018 | 3.9260 (2.5901 - 4.5141) |
| 2018-2020 | 9.6643 (5.4936 - 18.0015) |
| **55-64 years** |  |
| 1999-2018 | 3.7909 (2.5159 - 4.45) |
| 2018-2022 | 10.1192 (6.0369 - 18.3751) |
| **65-74 years** |  |
| 1999-2018 | 4.6364 (3.5589 - 5.3406) |
| 2018-2022 | 11.5317 (7.6731 - 19.3845) |
| **75-84 years** |  |
| 1999-2017 | 4.7446 (3.6082 - 5.6408) |
| 2017-2022 | 14.1864 (10.6995 - 20.8081) |
| **85+ years** |  |
| 1999-2018 | 3.6987 (2.7397 - 4.669) |
| 2018-2022 | 21.0773 (15.0582 - 30.5553) |

*For age groups, crude mortality rates were used for calculating APCs

**Supplemental Table 3: Overall and Sex‐Stratified** **CAD and Obesity-Related Age-Adjusted Mortality Rates per 100,000 in the United States, 1999 to 2022**

|  | **Age-Adjusted Rate (95% CI)** | | |
| --- | --- | --- | --- |
| **Year** | **Male** | **Female** | **Overall** |
| **1999** | 3.284 (3.16 - 3.409) | 2.54 (2.439 - 2.641) | 2.891 (2.812 - 2.97) |
| **2000** | 3.233 (3.111 - 3.355) | 2.464 (2.366 - 2.563) | 2.853 (2.775 - 2.931) |
| **2001** | 3.28 (3.158 - 3.401) | 2.413 (2.316 - 2.51) | 2.811 (2.735 - 2.887) |
| **2002** | 3.732 (3.604 - 3.86) | 2.7 (2.599 - 2.801) | 3.21 (3.129 - 3.291) |
| **2003** | 3.886 (3.757 - 4.015) | 2.66 (2.56 - 2.76) | 3.25 (3.17 - 3.331) |
| **2004** | 4.158 (4.025 - 4.291) | 2.934 (2.83 - 3.038) | 3.535 (3.452 - 3.619) |
| **2005** | 4.439 (4.303 - 4.575) | 2.927 (2.824 - 3.03) | 3.67 (3.585 - 3.754) |
| **2006** | 4.601 (4.464 - 4.738) | 3.001 (2.898 - 3.104) | 3.777 (3.692 - 3.862) |
| **2007** | 4.753 (4.615 - 4.892) | 2.944 (2.843 - 3.045) | 3.821 (3.737 - 3.906) |
| **2008** | 5.013 (4.873 - 5.154) | 3.064 (2.962 - 3.167) | 4.016 (3.93 - 4.103) |
| **2009** | 5.335 (5.191 - 5.479) | 3.204 (3.1 - 3.308) | 4.242 (4.154 - 4.33) |
| **2010** | 5.552 (5.406 - 5.697) | 3.25 (3.145 - 3.355) | 4.323 (4.236 - 4.411) |
| **2011** | 5.922 (5.773 - 6.071) | 3.425 (3.318 - 3.531) | 4.627 (4.537 - 4.718) |
| **2012** | 6.306 (6.154 - 6.458) | 3.64 (3.531 - 3.75) | 4.942 (4.849 - 5.035) |
| **2013** | 6.468 (6.315 - 6.621) | 3.648 (3.541 - 3.756) | 5.011 (4.919 - 5.104) |
| **2014** | 7.033 (6.875 - 7.191) | 3.909 (3.798 - 4.021) | 5.413 (5.317 - 5.508) |
| **2015** | 7.063 (6.906 - 7.221) | 3.877 (3.767 - 3.987) | 5.399 (5.304 - 5.493) |
| **2016** | 7.51 (7.348 - 7.672) | 4.056 (3.945 - 4.168) | 5.678 (5.582 - 5.774) |
| **2017** | 7.798 (7.635 - 7.961) | 4.132 (4.02 - 4.244) | 5.9 (5.803 - 5.998) |
| **2018** | 8.179 (8.013 - 8.345) | 4.108 (3.997 - 4.219) | 6.071 (5.973 - 6.17) |
| **2019** | 8.38 (8.212 - 8.547) | 4.338 (4.224 - 4.452) | 6.28 (6.18 - 6.38) |
| **2020** | 11.037 (10.846 - 11.227) | 5.66 (5.531 - 5.788) | 8.225 (8.112 - 8.338) |
| **2021** | 12.681 (12.477 - 12.884) | 6.66 (6.52 - 6.8) | 9.526 (9.405 - 9.647) |
| **2022** | 11.422 (11.231 - 11.614) | 6.059 (5.927 - 6.192) | 8.636 (8.521 - 8.751) |

**Supplemental Table 4: CAD and Obesity Related Age-Adjusted Mortality Rates per 100,000, Stratified by Race in the United States, 1999 to 2022**

|  | Age-Adjusted Rate (95% CI) | | | |
| --- | --- | --- | --- | --- |
| Year | **NH White** | **NH Black or African American** | **NH other** | **Hispanic or Latino** |
| 1999 | 2.897 (2.809 - 2.986) | 4.686 (4.357 - 5.015) | 0.695 (0.503 - 0.936) | Unreliable (0.222-0.592) |
| 2000 | 2.87 (2.783 - 2.958) | 4.166 (3.86 - 4.472) | 0.877 (0.657 - 1.147) | 0.400 (0.241-0.625) |
| 2001 | 2.888 (2.8 - 2.976) | 4.185 (3.884 - 4.487) | 0.659 (0.489 - 0.868) | 0.278 (0.167-0.434) |
| 2002 | 3.28 (3.187 - 3.373) | 4.74 (4.425 - 5.055) | 0.941 (0.732 - 1.191) | 0.507 (0.345-0.72) |
| 2003 | 3.369 (3.275 - 3.462) | 4.487 (4.182 - 4.792) | 1.154 (0.92 - 1.388) | 0.443 (0.305-0.623) |
| 2004 | 3.653 (3.556 - 3.75) | 5.085 (4.764 - 5.407) | 0.912 (0.722 - 1.137) | 0.526 (0.374-0.719) |
| 2005 | 3.762 (3.664 - 3.859) | 5.2 (4.88 - 5.519) | 1.461 (1.212 - 1.711) | 0.663 (0.492-0.875) |
| 2006 | 3.864 (3.766 - 3.963) | 5.256 (4.939 - 5.574) | 1.297 (1.071 - 1.523) | 0.532 (0.389-0.709) |
| 2007 | 3.989 (3.888 - 4.089) | 5.256 (4.942 - 5.57) | 1.317 (1.09 - 1.543) | 0.653 (0.493-0.848) |
| 2008 | 4.181 (4.079 - 4.282) | 5.453 (5.137 - 5.769) | 1.413 (1.184 - 1.642) | 0.696 (0.531-0.896) |
| 2009 | 4.383 (4.279 - 4.487) | 5.794 (5.473 - 6.115) | 1.497 (1.264 - 1.729) | 0.657 (0.505-0.841) |
| 2010 | 4.499 (4.394 - 4.604) | 5.827 (5.509 - 6.145) | 1.619 (1.382 - 1.856) | 0.733 (0.570-0.928) |
| 2011 | 4.869 (4.76 - 4.979) | 6.027 (5.709 - 6.346) | 1.657 (1.424 - 1.89) | 0.824 (0.657-1.020) |
| 2012 | 5.211 (5.098 - 5.323) | 6.445 (6.117 - 6.773) | 1.829 (1.589 - 2.068) | 0.898 (0.728-1.097) |
| 2013 | 5.253 (5.141 - 5.365) | 6.852 (6.516 - 7.187) | 1.552 (1.335 - 1.769) | 0.813 (0.653-1.000) |
| 2014 | 5.694 (5.578 - 5.81) | 7.158 (6.82 - 7.495) | 2.006 (1.763 - 2.248) | 0.925 (0.748-1.102) |
| 2015 | 5.679 (5.563 - 5.795) | 7.175 (6.841 - 7.508) | 2.015 (1.775 - 2.254) | 1.066 (0.878-1.254) |
| 2016 | 5.971 (5.852 - 6.09) | 7.924 (7.577 - 8.271) | 2.04 (1.809 - 2.272) | 0.938 (0.772-1.104) |
| 2017 | 6.274 (6.152 - 6.395) | 7.794 (7.453 - 8.134) | 2.023 (1.794 - 2.251) | 1.088 (0.907-1.268) |
| 2018 | 6.549 (6.425 - 6.673) | 7.585 (7.254 - 7.916) | 2.077 (1.851 - 2.304) | 1.139 (0.961-1.317) |
| 2019 | 6.655 (6.53 - 6.779) | 8.422 (8.073 - 8.77) | 2.166 (1.937 - 2.395) | 1.269 (1.082-1.455) |
| 2020 | 8.377 (8.238 - 8.516) | 11.816 (11.408 - 12.223) | 3.17 (2.896 - 3.443) | 1.656 (1.446-1.867) |
| 2021 | 10.084 (9.93 - 10.237) | 13.291 (12.852 - 13.729) | 3.301 (3.04 - 3.561) | 6.838 (6.546 - 7.131) |
| 2022 | 9.234 (9.088 - 9.38) | 11.867 (11.455 - 12.278) | 3.034 (2.787 - 3.281) | 5.616 (5.353 - 5.879) |

NH = non-Hispanic.

**Supplemental Table 5: CAD and Obesity Related Age-Adjusted Mortality Rates per 100,000, Stratified by States in the United States, 1999 to 2022**

| **State** | **Age-Adjusted Rate (95%CI)** | |
| --- | --- | --- |
|  | **1999-2020** | **2021-2022** |
| Alabama | 2.255 (2.146 - 2.363) | 5.453 (4.923 - 5.984) |
| Alaska | 4.868 (4.392 - 5.345) | 9.979 (7.949 - 12.009) |
| Arizona | 4.505 (4.371 - 4.64) | 9.835 (9.241 - 10.429) |
| Arkansas | 4.614 (4.411 - 4.816) | 11.114 (10.125 - 12.104) |
| California | 4.74 (4.681 - 4.798) | 7.065 (6.847 - 7.283) |
| Colorado | 5.865 (5.688 - 6.042) | 10.461 (9.771 - 11.151) |
| Connecticut | 2.956 (2.816 - 3.097) | 6.795 (6.104 - 7.486) |
| Delaware | 7.167 (6.731 - 7.604) | 14.556 (12.734 - 16.377) |
| District of Columbia | 5.721 (5.215 - 6.226) | 11.449 (9.187 - 13.712) |
| Florida | 3.771 (3.704 - 3.838) | 7.213 (6.933 - 7.493) |
| Georgia | 3.078 (2.984 - 3.173) | 7.777 (7.332 - 8.222) |
| Hawaii | 3.873 (3.607 - 4.138) | 5.358 (4.413 - 6.304) |
| Idaho | 4.985 (4.695 - 5.276) | 11.823 (10.552 - 13.095) |
| Illinois | 3.465 (3.382 - 3.549) | 5.874 (5.526 - 6.221) |
| Indiana | 5.054 (4.914 - 5.195) | 8.628 (8.06 - 9.195) |
| Iowa | 6.993 (6.759 - 7.227) | 14.335 (13.268 - 15.402) |
| Kansas | 5.395 (5.174 - 5.616) | 10.387 (9.404 - 11.369) |
| Kentucky | 4.956 (4.786 - 5.126) | 9.762 (9.016 - 10.508) |
| Louisiana | 4.699 (4.535 - 4.863) | 12.195 (11.348 - 13.043) |
| Maine | 4.543 (4.268 - 4.818) | 6.33 (5.319 - 7.342) |
| Maryland | 3.896 (3.766 - 4.026) | 8.18 (7.603 - 8.756) |
| Massachusetts | 2.965 (2.861 - 3.068) | 5.37 (4.927 - 5.813) |
| Michigan | 5.554 (5.437 - 5.67) | 8.903 (8.432 - 9.375) |
| Minnesota | 5.275 (5.118 - 5.433) | 10.619 (9.915 - 11.324) |
| Mississippi | 5.813 (5.585 - 6.041) | 10.286 (9.308 - 11.263) |
| Missouri | 3.784 (3.659 - 3.909) | 6.767 (6.237 - 7.298) |
| Montana | 5.325 (4.972 - 5.678) | 15.479 (13.631 - 17.328) |
| Nebraska | 3.654 (3.43 - 3.878) | 7.823 (6.806 - 8.841) |
| Nevada | 4.706 (4.493 - 4.919) | 10.588 (9.668 - 11.508) |
| New Hampshire | 4.756 (4.468 - 5.044) | 8.996 (7.771 - 10.222) |
| New Jersey | 3.843 (3.741 - 3.945) | 5.046 (4.682 - 5.411) |
| New Mexico | 5.547 (5.283 - 5.811) | 13.518 (12.238 - 14.799) |
| New York | 4.844 (4.766 - 4.922) | 8.753 (8.42 - 9.086) |
| North Carolina | 5.469 (5.349 - 5.59) | 7.826 (7.388 - 8.264) |
| North Dakota | 7.752 (7.219 - 8.285) | 14.09 (11.869 - 16.311) |
| Ohio | 5.514 (5.406 - 5.621) | 9.612 (9.154 - 10.07) |
| Oklahoma | 8.941 (8.694 - 9.189) | 17.181 (16.11 - 18.252) |
| Oregon | 4.822 (4.649 - 4.995) | 7.708 (7.057 - 8.359) |
| Pennsylvania | 4.331 (4.242 - 4.42) | 8.632 (8.231 - 9.034) |
| Rhode Island | 6.276 (5.899 - 6.653) | 15.476 (13.64 - 17.313) |
| South Carolina | 4.518 (4.363 - 4.673) | 18.546 (17.617 - 19.475) |
| South Dakota | 5.61 (5.201 - 6.019) | 11.088 (9.308 - 12.868) |
| Tennessee | 5.818 (5.667 - 5.969) | 12.434 (11.75 - 13.118) |
| Texas | 5.056 (4.98 - 5.132) | 11.084 (10.756 - 11.412) |
| Utah | 3.731 (3.519 - 3.944) | 8.906 (7.972 - 9.84) |
| Vermont | 16.02 (15.26 - 16.78) | 30.68 (27.448 - 33.911) |
| Virginia | 2.866 (2.77 - 2.963) | 5.575 (5.167 - 5.982) |
| Washington | 4.936 (4.8 - 5.072) | 10.871 (10.271 - 11.47) |
| West Virginia | 6.859 (6.56 - 7.159) | 14.451 (13.053 - 15.849) |
| Wisconsin | 7.224 (7.049 - 7.399) | 17.137 (16.294 - 17.98) |
| Wyoming | 7.086 (6.516 - 7.655) | 16.959 (14.293 - 19.625) |

**Supplemental Table 6: CAD and Obesity Related Age-Adjusted Mortality Rates per 100,000, Stratified by Census Region in the United States, 1999 to 2022**

| **Census Region** | **Year** | **Age-Adjusted Rate (95% CI)** |
| --- | --- | --- |
| **Northeast** |  |  |
| **Northeast** | 1999 | 2.929 (2.752 - 3.106) |
| **Northeast** | 2000 | 2.744 (2.574 - 2.915) |
| **Northeast** | 2001 | 2.665 (2.498 - 2.832) |
| **Northeast** | 2002 | 2.919 (2.745 - 3.093) |
| **Northeast** | 2003 | 3.008 (2.833 - 3.182) |
| **Northeast** | 2004 | 3.301 (3.119 - 3.484) |
| **Northeast** | 2005 | 3.222 (3.043 - 3.402) |
| **Northeast** | 2006 | 3.308 (3.128 - 3.488) |
| **Northeast** | 2007 | 3.762 (3.57 - 3.955) |
| **Northeast** | 2008 | 3.717 (3.527 - 3.907) |
| **Northeast** | 2009 | 3.854 (3.662 - 4.046) |
| **Northeast** | 2010 | 4.198 (3.999 - 4.398) |
| **Northeast** | 2011 | 4.27 (4.069 - 4.47) |
| **Northeast** | 2012 | 4.598 (4.391 - 4.805) |
| **Northeast** | 2013 | 4.742 (4.533 - 4.951) |
| **Northeast** | 2014 | 4.893 (4.682 - 5.104) |
| **Northeast** | 2015 | 5.25 (5.032 - 5.468) |
| **Northeast** | 2016 | 5.57 (5.346 - 5.794) |
| **Northeast** | 2017 | 5.643 (5.419 - 5.866) |
| **Northeast** | 2018 | 5.751 (5.525 - 5.977) |
| **Northeast** | 2019 | 6.126 (5.89 - 6.361) |
| **Northeast** | 2020 | 7.868 (7.603 - 8.133) |
| **Northeast** | 2021 | 8.154 (7.89 - 8.418) |
| **Northeast** | 2022 | 7.694 (7.437 - 7.951) |
| **Midwest** |  |  |
| **Midwest** | 1999 | 2.985 (2.818 - 3.152) |
| **Midwest** | 2000 | 2.954 (2.788 - 3.12) |
| **Midwest** | 2001 | 3.01 (2.845 - 3.175) |
| **Midwest** | 2002 | 3.336 (3.164 - 3.508) |
| **Midwest** | 2003 | 3.353 (3.181 - 3.526) |
| **Midwest** | 2004 | 3.759 (3.579 - 3.94) |
| **Midwest** | 2005 | 3.817 (3.635 - 3.999) |
| **Midwest** | 2006 | 3.73 (3.552 - 3.907) |
| **Midwest** | 2007 | 3.856 (3.677 - 4.035) |
| **Midwest** | 2008 | 4.258 (4.071 - 4.446) |
| **Midwest** | 2009 | 4.302 (4.114 - 4.49) |
| **Midwest** | 2010 | 4.586 (4.392 - 4.78) |
| **Midwest** | 2011 | 5.136 (4.931 - 5.34) |
| **Midwest** | 2012 | 5.716 (5.501 - 5.931) |
| **Midwest** | 2013 | 5.76 (5.545 - 5.975) |
| **Midwest** | 2014 | 6.43 (6.205 - 6.655) |
| **Midwest** | 2015 | 6.243 (6.023 - 6.464) |
| **Midwest** | 2016 | 6.583 (6.356 - 6.81) |
| **Midwest** | 2017 | 6.815 (6.586 - 7.043) |
| **Midwest** | 2018 | 7.116 (6.883 - 7.349) |
| **Midwest** | 2019 | 7.098 (6.867 - 7.329) |
| **Midwest** | 2020 | 9.007 (8.749 - 9.266) |
| **Midwest** | 2021 | 10.027 (9.753 - 10.301) |
| **Midwest** | 2022 | 8.954 (8.696 - 9.211) |
| **South** |  |  |
| **South** | 1999 | 2.705 (2.577 - 2.834) |
| **South** | 2000 | 2.753 (2.624 - 2.881) |
| **South** | 2001 | 2.719 (2.593 - 2.845) |
| **South** | 2002 | 3.078 (2.945 - 3.21) |
| **South** | 2003 | 3.167 (3.034 - 3.3) |
| **South** | 2004 | 3.426 (3.289 - 3.563) |
| **South** | 2005 | 3.604 (3.465 - 3.742) |
| **South** | 2006 | 3.867 (3.724 - 4.009) |
| **South** | 2007 | 3.761 (3.622 - 3.9) |
| **South** | 2008 | 3.81 (3.672 - 3.948) |
| **South** | 2009 | 4.284 (4.138 - 4.429) |
| **South** | 2010 | 4.281 (4.137 - 4.425) |
| **South** | 2011 | 4.342 (4.198 - 4.486) |
| **South** | 2012 | 4.606 (4.459 - 4.753) |
| **South** | 2013 | 4.696 (4.55 - 4.842) |
| **South** | 2014 | 5.244 (5.09 - 5.399) |
| **South** | 2015 | 5.051 (4.901 - 5.201) |
| **South** | 2016 | 5.314 (5.161 - 5.467) |
| **South** | 2017 | 5.526 (5.372 - 5.68) |
| **South** | 2018 | 5.666 (5.511 - 5.82) |
| **South** | 2019 | 6.07 (5.91 - 6.229) |
| **South** | 2020 | 8.224 (8.04 - 8.407) |
| **South** | 2021 | 10.127 (9.923 - 10.331) |
| **South** | 2022 | 9.224 (9.032 - 9.417) |
| **West** |  |  |
| **West** | 1999 | 3.25 (3.066 - 3.435) |
| **West** | 2000 | 3.145 (2.965 - 3.325) |
| **West** | 2001 | 3.035 (2.862 - 3.209) |
| **West** | 2002 | 3.595 (3.409 - 3.781) |
| **West** | 2003 | 3.614 (3.429 - 3.799) |
| **West** | 2004 | 3.723 (3.537 - 3.91) |
| **West** | 2005 | 4.018 (3.828 - 4.207) |
| **West** | 2006 | 4.135 (3.943 - 4.326) |
| **West** | 2007 | 3.937 (3.753 - 4.12) |
| **West** | 2008 | 4.293 (4.103 - 4.483) |
| **West** | 2009 | 4.423 (4.233 - 4.613) |
| **West** | 2010 | 4.307 (4.12 - 4.493) |
| **West** | 2011 | 4.917 (4.72 - 5.115) |
| **West** | 2012 | 5.019 (4.823 - 5.215) |
| **West** | 2013 | 5.022 (4.828 - 5.216) |
| **West** | 2014 | 5.141 (4.947 - 5.335) |
| **West** | 2015 | 5.386 (5.188 - 5.584) |
| **West** | 2016 | 5.621 (5.422 - 5.82) |
| **West** | 2017 | 5.826 (5.624 - 6.027) |
| **West** | 2018 | 5.994 (5.792 - 6.197) |
| **West** | 2019 | 5.987 (5.785 - 6.188) |
| **West** | 2020 | 7.813 (7.584 - 8.041) |
| **West** | 2021 | 9.24 (8.991 - 9.488) |
| **West** | 2022 | 8.143 (7.913 - 8.374) |

**Supplemental Table 7: CAD and Obesity Related Age-Adjusted Mortality Rates per 100,000 in United States stratified by Urban-Rural Classification, 1999-2020**

|  | **Age-Adjusted Rate (95% CI)** | |
| --- | --- | --- |
| **Year** | **Urban** | **Rural** |
| 1999 | 2.849 (2.763 - 2.936) | 3.165 (2.967 - 3.362) |
| 2000 | 2.777 (2.692 - 2.862) | 3.423 (3.218 - 3.628) |
| 2001 | 2.753 (2.669 - 2.836) | 3.295 (3.096 - 3.495) |
| 2002 | 3.066 (2.979 - 3.153) | 3.98 (3.761 - 4.198) |
| 2003 | 3.087 (3.001 - 3.174) | 4.15 (3.928 - 4.372) |
| 2004 | 3.39 (3.299 - 3.48) | 4.274 (4.051 - 4.497) |
| 2005 | 3.445 (3.355 - 3.534) | 4.694 (4.461 - 4.927) |
| 2006 | 3.562 (3.472 - 3.652) | 4.79 (4.555 - 5.024) |
| 2007 | 3.671 (3.58 - 3.762) | 4.71 (4.478 - 4.942) |
| 2008 | 3.803 (3.711 - 3.894) | 5.001 (4.765 - 5.238) |
| 2009 | 4.014 (3.921 - 4.108) | 5.341 (5.098 - 5.585) |
| 2010 | 4.117 (4.023 - 4.211) | 5.446 (5.202 - 5.689) |
| 2011 | 4.373 (4.277 - 4.469) | 6.022 (5.762 - 6.282) |
| 2012 | 4.623 (4.526 - 4.721) | 6.531 (6.26 - 6.803) |
| 2013 | 4.758 (4.659 - 4.856) | 6.416 (6.148 - 6.685) |
| 2014 | 5.119 (5.018 - 5.22) | 7.029 (6.748 - 7.31) |
| 2015 | 5.133 (5.033 - 5.234) | 7.075 (6.797 - 7.353) |
| 2016 | 5.396 (5.294 - 5.498) | 7.445 (7.156 - 7.733) |
| 2017 | 5.557 (5.454 - 5.66) | 7.809 (7.516 - 8.103) |
| 2018 | 5.703 (5.599 - 5.806) | 8.085 (7.788 - 8.383) |
| 2019 | 5.845 (5.741 - 5.95) | 8.658 (8.35 - 8.965) |
| 2020 | 7.79 (7.671 - 7.909) | 10.809 (10.467 - 11.15) |

**Supplementary Figure 1: Coronary artery disease and Obesity-Related Annual Percentage Change (APC) in the United States from 1999 to 2022 stratified by (A) Census Region (B) Sex (C) Race and (D) Urbanization**

**A)
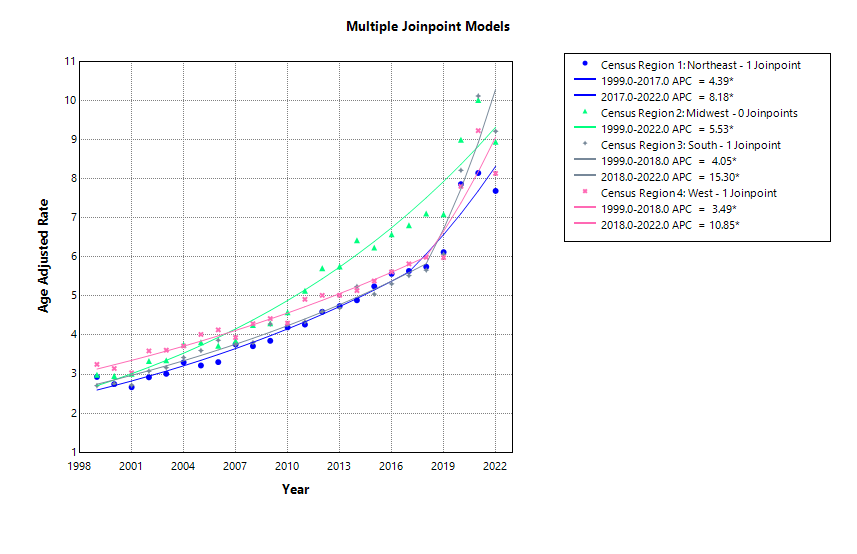
**

**B)
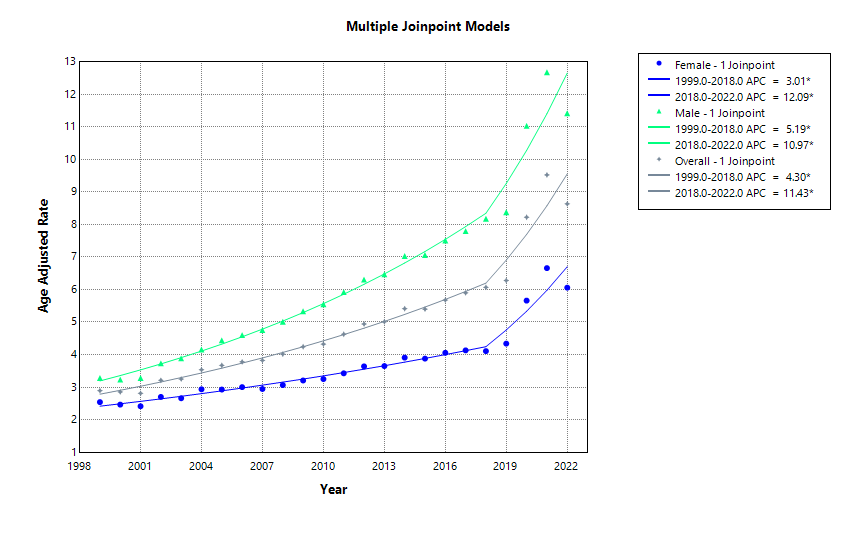
**

**C)
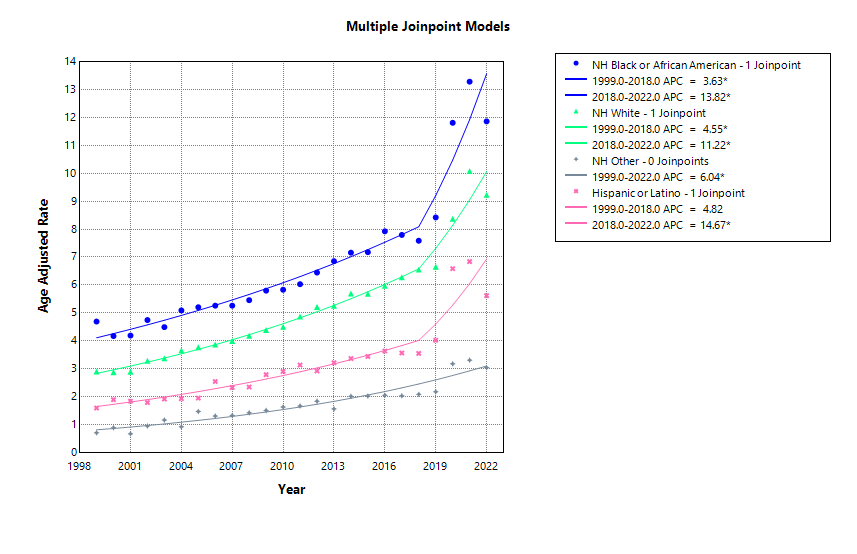
**

**D)
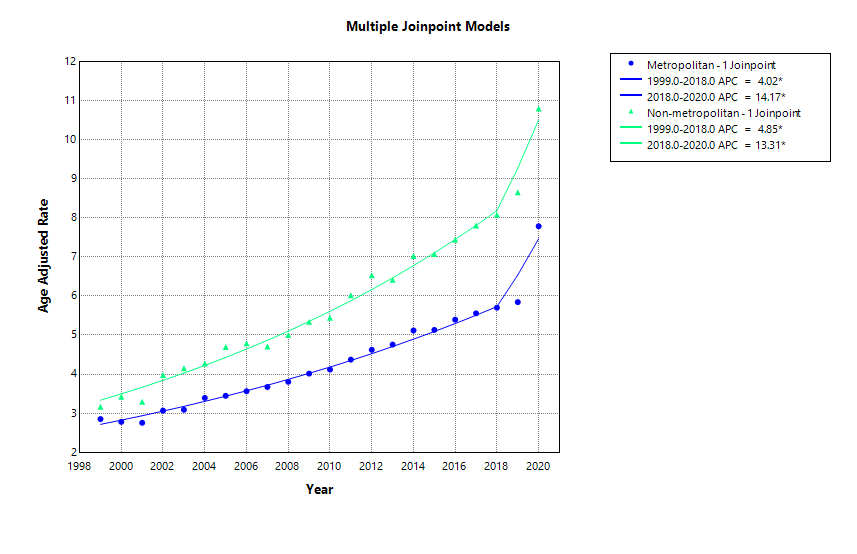
**

*****Data for urbanization is available only from 1999-2020

**Supplementary Figure 2: Coronary artery disease and Obesity-Related Annual Percentage Change in the United States from 1999 to 2022 stratified by 10-year age groups**

**
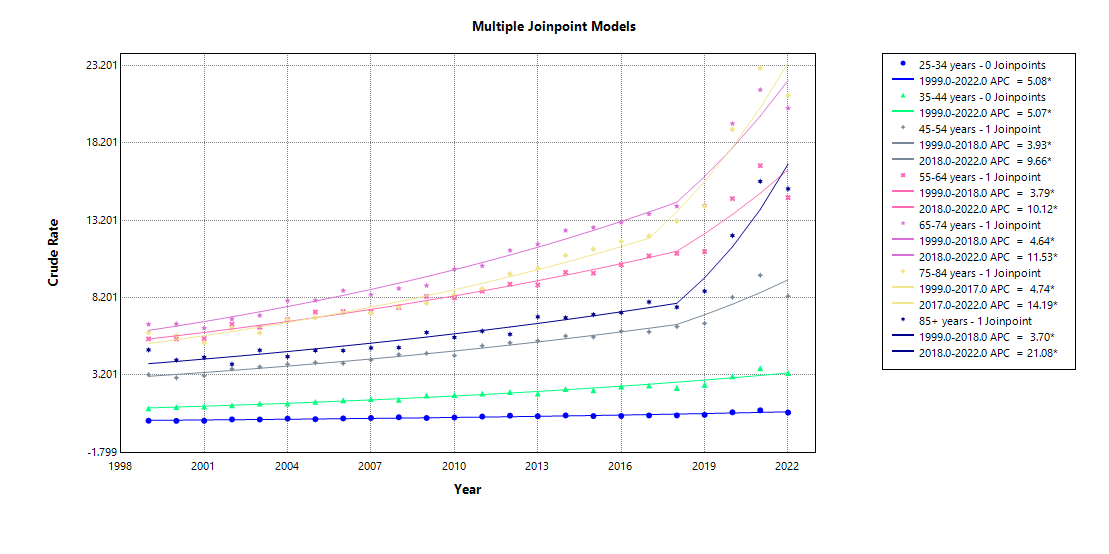
**

**Supplemental Table 8: Pairwise comparison of different subgroups stratified by (sex, race, census region, and urbanization)**

| **Cohort 1** | **Cohort 2** | **P-Value** |
| --- | --- | --- |
| **Overall and Gender** | | |
| Female | Male | 0.000222 |
| Female | Overall | 0.000222 |
| Male | Overall | 0.000222 |
| **Census Region** | | |
| Northeast | Midwest | 0.094444 |
| Northeast | South | 0.006 |
| Northeast | West | 0.001333 |
| Midwest | South | 0.000667 |
| Midwest | West | 0.000222 |
| South | West | 0.000222 |
| **Race** | | |
| NH Black or African American | | 0.024222 |
| NH Black or African American | NH Other | 0.236 |
| NH Black or African American | NH White | 0.004889 |
| Hispanic or Latino | NH Other | 0.799778 |
| Hispanic or Latino | NH White | 0.148667 |
| NH Other | NH White | 0.309556 |
| **Urbanization** | | |
| Urban | Rural | 0.000444 |

**Supplemental Table 9: Coronary Artery Disease and Obesity-Related Crude Mortality Rates (CMR) per 100,000 individuals Stratified by Ten Year Age Groups in the United States, 1999 to 2022**

|  | **Crude Mortality Rates (95% CI)** | | | | | | |
| --- | --- | --- | --- | --- | --- | --- | --- |
| **Year** | **25-34 Year** | **35-44 Year** | **45-54** | **55-64** | **65-74** | **75-84** | **85+** |
| **1999** | 0.236 (0.191 - 0.289) | 1.034 (0.94 - 1.128) | 3.215 (3.031 - 3.399) | 5.522 (5.223 - 5.821) | 6.472 (6.104 - 6.839) | 5.939 (5.507 - 6.371) | 4.815 (4.147 - 5.482) |
| **2000** | 0.228 (0.184 - 0.28) | 1.121 (1.023 - 1.218) | 3.015 (2.84 - 3.19) | 5.528 (5.233 - 5.824) | 6.492 (6.124 - 6.861) | 5.752 (5.329 - 6.175) | 4.151 (3.538 - 4.765) |
| **2001** | 0.233 (0.188 - 0.286) | 1.15 (1.051 - 1.249) | 3.138 (2.963 - 3.313) | 5.549 (5.257 - 5.84) | 6.223 (5.862 - 6.583) | 5.273 (4.871 - 5.674) | 4.336 (3.715 - 4.958) |
| **2002** | 0.325 (0.269 - 0.382) | 1.23 (1.127 - 1.333) | 3.581 (3.395 - 3.766) | 6.475 (6.17 - 6.78) | 6.803 (6.426 - 7.18) | 6.236 (5.803 - 6.669) | 3.891 (3.306 - 4.476) |
| **2003** | 0.316 (0.26 - 0.372) | 1.341 (1.233 - 1.449) | 3.714 (3.527 - 3.901) | 6.277 (5.983 - 6.57) | 7.043 (6.66 - 7.425) | 5.909 (5.489 - 6.328) | 4.792 (4.15 - 5.434) |
| **2004** | 0.374 (0.314 - 0.435) | 1.342 (1.234 - 1.451) | 3.894 (3.704 - 4.083) | 6.774 (6.476 - 7.071) | 7.998 (7.592 - 8.404) | 6.767 (6.319 - 7.214) | 4.378 (3.769 - 4.986) |
| **2005** | 0.336 (0.279 - 0.394) | 1.455 (1.342 - 1.568) | 3.996 (3.806 - 4.186) | 7.265 (6.963 - 7.566) | 8.018 (7.614 - 8.422) | 6.891 (6.441 - 7.341) | 4.773 (4.148 - 5.398) |
| **2006** | 0.383 (0.322 - 0.444) | 1.563 (1.445 - 1.681) | 3.944 (3.756 - 4.131) | 7.3 (7.004 - 7.597) | 8.65 (8.234 - 9.066) | 7.224 (6.764 - 7.684) | 4.768 (4.154 - 5.381) |
| **2007** | 0.413 (0.35 - 0.476) | 1.636 (1.514 - 1.757) | 4.169 (3.978 - 4.36) | 7.293 (7.002 - 7.584) | 8.381 (7.977 - 8.786) | 7.19 (6.731 - 7.65) | 4.941 (4.327 - 5.555) |
| **2008** | 0.46 (0.394 - 0.526) | 1.586 (1.465 - 1.706) | 4.51 (4.312 - 4.707) | 7.556 (7.265 - 7.848) | 8.788 (8.382 - 9.194) | 7.609 (7.136 - 8.082) | 4.966 (4.36 - 5.571) |
| **2009** | 0.413 (0.35 - 0.475) | 1.868 (1.737 - 2) | 4.591 (4.393 - 4.79) | 8.273 (7.973 - 8.572) | 8.977 (8.574 - 9.38) | 7.832 (7.352 - 8.313) | 5.943 (5.291 - 6.596) |
| **2010** | 0.446 (0.381 - 0.51) | 1.899 (1.766 - 2.032) | 4.444 (4.249 - 4.639) | 8.193 (7.899 - 8.487) | 10.044 (9.623 - 10.466) | 8.376 (7.88 - 8.872) | 5.625 (4.998 - 6.252) |
| **2011** | 0.505 (0.437 - 0.573) | 1.994 (1.856 - 2.131) | 5.085 (4.876 - 5.294) | 8.615 (8.32 - 8.91) | 10.248 (9.83 - 10.667) | 8.766 (8.261 - 9.272) | 6.031 (5.395 - 6.666) |
| **2012** | 0.563 (0.491 - 0.634) | 2.095 (1.954 - 2.236) | 5.275 (5.061 - 5.489) | 9.065 (8.765 - 9.366) | 11.249 (10.824 - 11.673) | 9.727 (9.196 - 10.257) | 5.826 (5.209 - 6.443) |
| **2013** | 0.53 (0.461 - 0.599) | 1.988 (1.85 - 2.125) | 5.408 (5.19 - 5.626) | 9.019 (8.722 - 9.316) | 11.655 (11.234 - 12.076) | 10.099 (9.562 - 10.636) | 6.953 (6.288 - 7.618) |
| **2014** | 0.579 (0.508 - 0.651) | 2.288 (2.141 - 2.435) | 5.713 (5.489 - 5.938) | 9.831 (9.524 - 10.138) | 12.539 (12.112 - 12.966) | 10.926 (10.372 - 11.48) | 6.897 (6.241 - 7.553) |
| **2015** | 0.537 (0.469 - 0.605) | 2.198 (2.053 - 2.342) | 5.645 (5.421 - 5.869) | 9.778 (9.475 - 10.081) | 12.733 (12.312 - 13.154) | 11.326 (10.767 - 11.885) | 7.094 (6.435 - 7.752) |
| **2016** | 0.544 (0.476 - 0.612) | 2.449 (2.296 - 2.601) | 6.025 (5.793 - 6.258) | 10.327 (10.018 - 10.637) | 13.063 (12.644 - 13.482) | 11.831 (11.266 - 12.396) | 7.225 (6.566 - 7.885) |
| **2017** | 0.573 (0.504 - 0.643) | 2.508 (2.354 - 2.661) | 5.982 (5.749 - 6.215) | 10.899 (10.583 - 11.214) | 13.604 (13.184 - 14.023) | 12.178 (11.614 - 12.742) | 7.915 (7.229 - 8.601) |
| **2018** | 0.58 (0.51 - 0.65) | 2.357 (2.209 - 2.505) | 6.32 (6.078 - 6.561) | 11.054 (10.737 - 11.371) | 14.102 (13.68 - 14.523) | 13.148 (12.575 - 13.72) | 7.579 (6.912 - 8.246) |
| **2019** | 0.614 (0.542 - 0.685) | 2.566 (2.412 - 2.72) | 6.532 (6.284 - 6.78) | 11.171 (10.853 - 11.489) | 14.169 (13.754 - 14.585) | 14.108 (13.525 - 14.69) | 8.615 (7.907 - 9.323) |
| **2020** | 0.779 (0.699 - 0.86) | 3.109 (2.941 - 3.277) | 8.222 (7.943 - 8.502) | 14.598 (14.234 - 14.961) | 19.447 (18.968 - 19.926) | 19.074 (18.407 - 19.742) | 12.21 (11.371 - 13.049) |
| **2021** | 0.91 (0.822 - 0.998) | 3.631 (3.452 - 3.81) | 9.639 (9.337 - 9.941) | 16.73 (16.343 - 17.118) | 21.633 (21.136 - 22.13) | 23.035 (22.296 - 23.774) | 15.713 (14.708 - 16.719) |
| **2022** | 0.763 (0.682 - 0.843) | 3.321 (3.15 - 3.492) | 8.291 (8.01 - 8.571) | 14.665 (14.3 - 15.031) | 20.445 (19.963 - 20.927) | 21.261 (20.578 - 21.944) | 15.233 (14.283 - 16.183) |
